# 

**Published:** 1879-02

**Authors:** 


					﻿Whole Number,
CCCCV.
February, 1879.
Number 2,
Vol. XXXVIII.
THE
CHICAGO
M JT1) L CAL
J ournal and Examiner
EDITORS:
WILLIAM H. BYFORD, A.M., M.D..
JAS. NEVINS HYDE, A.M., M.D. FERD. C. HOTZ, M.D.
E. FLETCHER INCALS, M.D.
CHICAGO:
PUBLISHED BY TIIE CHICAGO MEDICAL PRESS ASSOCIATION.
188 Clark Street.
3“Read the Notice of this Journal among Advertisements.
				

